# Supplementary material for: Reconciling Mining with the Conservation of Cave Biodiversity: A Quantitative Baseline to Help Establish Conservation Priorities
Source: PLoS One. 2016 Dec 20;11(12):e0168348. doi: 10.1371/journal.pone.0168348 (PMC5173368; doi:10.1371/journal.pone.0168348)
Supplement: S1 Dataset — (ZIP) [file pone.0168348.s002.zip › Taxa/Serra Norte/SN_2013/N4WS/Lista N4WS-60.pdf]

CAVIDADE N4WS-0060

Classe	Ordem	Fam/Outros	Gên/Outros	Espécie	S-U	U-S	Zon.
Arachnida	Acari	Bdellidae		sp.2		X	P
Arachnida	Amblypygi	Charinidae	*Charinus*	jovem		2	P
Arachnida	Amblypygi	Phrynidae	*Heterophrynus*	*longicornis*	1		E
Arachnida	Araneae	Corinnidae		sp.1	1		E
Arachnida	Araneae	Ochyroceratidae	*Ochyrocera*	sp.1	X	X	P
Arachnida	Araneae	Oonopidae		jovem		X	P
Arachnida	Araneae	Pholcidae	Ninetinae	sp.1		X	P
Arachnida	Araneae	Scytodidae	*Scytodes*	jovem	1		E
Arachnida	Araneae	Theridiidae		jovem		X	P
Arachnida	Opiliones	Cosmetidae		jovem		1	P
Arachnida	Opiliones	Escadabiidae		sp.1		X	P
Arachnida	Pseudoscorpiones	Chernetidae	*Spelaeochernes*	sp.1		X	P
Entognatha	Diplura	Campodeidae		sp.1		X	P
Insecta	Blattodea	Polyphagidae		jovem	1		E
Insecta	Coleoptera	Carabidae		jovem	X		E
Insecta	Coleoptera	Staphylinidae		sp.72	X		E
Insecta	Diptera	Culicidae	*Culex*	sp.	X		E
Insecta	Diptera	Psychodidae	*Evandromyia*	*saulensis*	X		E
Insecta	Diptera	Psychodidae	*Micropygomyia*	*pilosa*		X	P
Insecta	Diptera	Psychodidae	*Sciopemyia*	*sordellii*		X	E/P
Insecta	Diptera	Tipulidae	Tipulinae	sp.	X		E
Insecta	Heteroptera	Reduviidae	Triatominae	jovem		1	P
Insecta	Homoptera	Cixiidae		jovem		X	P
Insecta	Hymenoptera	Formicidae	*Camponotus*	sp.1		X	P
Insecta	Hymenoptera	Formicidae	*Nylanderia*	sp.2		X	P
Insecta	Hymenoptera	Formicidae	*Octostruma*	sp.1		X	P
Insecta	Hymenoptera	Formicidae	*Pachycondyla*	*striata*	X		E
Insecta	Hymenoptera	Formicidae	*Trachymyrmex*	sp.2		X	P
Insecta	Lepidoptera	Noctuoidea		sp.1	1		E
Amphibia	Anura	Strabomantidae	*Pristimantis*	cf. *fenestratus*	2	1	E/P
Mammalia	Chiroptera			sp.		1	P
